# Burnout and associated factors among Tunisian medical interns and residents

**DOI:** 10.1192/j.eurpsy.2022.1567

**Published:** 2022-09-01

**Authors:** A. Zouari, F. Guermazi, F. Tabib, M. Ben Abdallah, S. Hentati, I. Baati, J. Masmoudi

**Affiliations:** HEDI CHAKER HOSPITAL, Psychiatry A, sfax, Tunisia

**Keywords:** medical, intern, resident, burnout

## Abstract

**Introduction:**

Burnout is an occupational psychological syndrome induced by chronic stress defined by three dimensions: emotional exhaustion (EE), depersonalization (DP) and reduced personal accomplishment (PA).

**Objectives:**

Estimate burnout among residents and interns in Tunisia. Identify factors related to burnout.

**Methods:**

We conducted a cross-sectional, descriptive, and analytical study between March 1 and April 15, 2021. Data collection among young physicians was done by a self-questionnaire published online. The assessment of the degree of burnout was done by the Maslach Burnout Inventory (MBI).

**Results:**

The total number of participants was 56 of which 71.4% were women. The average age was 26.76 years. The pourcentage of the married was 21.4% of which 58.3% had children. 30.4% had parents in charge. Most of the participants worked in university hospitals and 75% of them in a medical department. Residents represented 64.3% of the participants. Number of working hours exceeded 40 hours per week in 60.7% of the cases with an average number of shifts per month estimated at 4.71±2.36. According to MBI, 94.6% of the participants had a score in favor of burnout, of which 19.6% was severe. The number of hours worked per week and the number of shifts per month were significantly associated with the presence of a burnout syndrome with respective correlation factors of 0.027 and 0.047.

**Conclusions:**

Most residents and interns suffered from burnout with a variable degree of severity. The workload with a greater number of working hours and on-call duty favored the emergence of this burnout.

**Disclosure:**

No significant relationships.

